# Supporting healthcare workers in times of COVID-19 with eye movement desensitization and reprocessing online: A pilot study

**DOI:** 10.3389/fpsyg.2022.964407

**Published:** 2022-08-08

**Authors:** Elisa Faretta, M. Ignazia Garau, Eugenio Gallina, Marco Pagani, Isabel Fernandez

**Affiliations:** ^1^Eye Movement Desensitization and Reprocessing (EMDR) Italy Association, Varedo, Italy; ^2^Centro di Ricerca e Studi in Psicotraumatologia (CRSP), Milan, Italy; ^3^Institute of Cognitive Sciences and Technologies, Consiglio Nazionale delle Ricerche, Rome, Italy

**Keywords:** healthcare workers, EMDR, PTSD, COVID-19, online

## Abstract

We report the results of a pilot study regarding the adaptation of the group eye movement desensitization and reprocessing (EMDR) protocol for the treatment online, for the management of trauma associated with the COVID-19 Pandemic in Italy. The target group were healthcare workers in a nursing home (Residenza sanitaria assistita, RSA) who decided to live and stay on site during the most acute phase of the Pandemic in order to protect the residents of the home. Scores for perceived post traumatic stress disorder (PTSD) symptoms and quality of emotional experience improved significantly following participation in the therapy programme. These preliminary results confirm the innovative potential of the EMDR protocol when used online on early intervention, to prevent the development of later psychological disturbances.

## Introduction

The COVID-19 Pandemic undoubtedly qualifies as a mass disaster, with significant negative effects on the social, cognitive and emotional functioning of everyone involved. One group that has been at the frontline throughout are health workers, who have had to deal with the emergency in hospitals and emergency services, in prevention and epidemiology services and in nursing homes (Residenza sanitaria assistita, RSA). These workers have been in a constant state of high physical and psychological alert (Johnson et al., 2020): emergencies produce a state of activation that enables rapid rescue attempts. Unsurprisingly, then, therapy experiences with this category of workers have evidenced emotional reactions that include fear, anxiety, and anger, aggravated and complicated by objective, subjective and organizational risk factors. While this is true in general of health field workers, it is a particularly acute problem for health workers operating in nursing homes (RSA). The residents are elderly, weak or vulnerable and need help and support with physical, emotional, relational, and medical/health needs, and it was in these structures that the sudden explosion of the epidemic was most overwhelming. The elderly were the population most affected by the pandemic as well as healthcare workers. Along with hospitals, nursing homes were identified by the health authorities as the main locus of spread of the COVID-19 Pandemic, making them the epicenter of this silent slaughter. As soon as the pandemic hit, nursing home workers were exposed to continuous cumulative traumatic experiences that resulted in repeated activation of extreme stress reactions. Each new activation further sensitized this population, increasing their risk of developing post traumatic stress disorder (PTSD) and other comorbid disturbances.

Of the therapeutic options available for the treatment of PTSD, eye movement desensitization and reprocessing (EMDR) is one of those that has been demonstrated to be highly effective (Greenberg et al., 2015). It is based on the adaptive information processing (AIP) model, according to which many psychological disorders are the manifestation of unresolved traumatic or stressful memories (Shapiro, 1995, 2001). One of the most important aspects of this therapy is the identification of the life events that have been particularly traumatic for the person (Poon, 2012).

The current emergency leads to the use of a group EMDR protocol, as has already been done in many countries worldwide following mass disasters. Group EMDR has been used both on adults and on children and has been adapted in many cases to suit specific contexts and circumstances (Wilson et al., 2000; Korkmazlar-Oral and Pamuk, 2002; Fernandez, 2007; Gelbach and Davis, 2007; Zaghrout-Hodali et al., 2008). The group protocol (EMDR-IGTP) combines standard 8-phase EMDR therapy with a group therapy model (Jarero et al., 1999; Artigas et al., 2000) and uses of a form of bilateral stimulation called the Butterfly Hug (Artigas and Jarero, 2014; Jarero, 2020) along with drawing tasks (Maxfield, 2008). Given the benefits of group therapy and the proven effectiveness of EMDR Integrative Group Treatment Protocol (EMDR-IGTP), we tested an online adaptation of the group EMDR protocol intended to respond to the needs generated by the current emergency, as also reported in recent literature (Luber, 2020; Pérez et al., 2020; Tarquinio et al., 2020; Fisher, 2021).

The study was conducted in a nursing home for the elderly where the healthcare staff had decided to live inside the facility in order to protect the residents from infection of COVID-19. Not only were the healthcare staff exposed to the risk of infection and to exhausting shifts, hard physical work and a reduction in personnel resulting in an emotional overload, but they had to bear these burdens without the support and presence of their families. Other complexities arose due to the measures necessary to prevent the spread of the virus, which lead to changes in the nursing home routines and restricted contact between residents and their families and between healthcare workers and their families.

The goals were to further test the feasibility of online group EMDR treatment and to qualitatively and quantitively assess its effects on the emotional and psychological health of participants. The expected outcomes, in line with these goals, pertained to two main areas: prevention of the chronicization of psychological disturbances; and strengthening of protective psychological resources.

## Materials and methods

### Research context

Participants were healthcare workers employed at a nursing home in Capoterra (CA). They had chosen to isolate themselves within the facility for 30 days in April, 2020 in order to avoid outside contact and the potential consequent infection of residents with COVID-19: it was well-established by then that therapeutic communities were among the hardest hit by the COVID-19 emergency. The owning company provided live-in accommodation to staff and the manager of the facility contacted the Italian EMDR association to request an intervention to support the operative team over this period.

The intervention was implemented online, and two groups were held by trained EMDR therapists whose job was both to evaluate stress levels and to help reprocess the disturbing experiences and promote self-protective resources.

### Participants

There were 11 participants: 7 women and 4 men with an average age of 46, divided into two groups therapy of 6 and 5 participants, respectively. All of them had chosen to isolate within the nursing home for the 30-day period from April 1 to April 30, 2020.

### Procedure

Two programmes of group therapy were carried out. Each consisted of five meetings: an initial pre-test and psychoeducational meeting, followed by three EMDR group therapy meetings, and a final post-test and debriefing meeting.

The interventions were conducted by therapists trained in EMDR, at a Consultant level (certified by EMDR Europe Association). All the meetings were carried out on a virtual conference platform to preserve social distancing. The aim of the project was to provide support to the care team to help them bear the emotional burden of isolation, to reduce stress and to contain anxiety and anger. This was done by offering a space where they could “describe” their most stressful experience, their negative feelings and process them through EMDR, in order to reduce their risk of developing post-traumatic stress.

The first meeting (meeting 1) in each intervention was dedicated to introducing the participants, presenting the project and conducing an initial pre-test of emotion and post-traumatic stress levels (IES-R scale and emotion thermometer) and a Critical Incident Stress Orientation (CISO, Maslovaric, 2020). Patients were informed about the confidentiality agreement.

Since the goal of the intervention was to improve participants’ psychological wellbeing, initial screening of their symptoms in the acute phase was carried out.

The CISO has the following features:

• It provides a symptom grid showing Normal and Common reactions to post-traumatic stress. This allows participants to identify their own symptoms as common, albeit disturbing. It also provides explanations and descriptions of what is meant by a traumatic event, the psychological reactions that can arise following one, and vulnerability factors (in this case level of absurdity and level of involvement);
• It provides indications for emotional self-protection to support coping strategies and resilience;
• It provides explanations of the EMDR approach, and in particular on the short group protocol (EMDR-IGTP).

Accordingly, the CISO phase of meeting 1 started with normalization of participants’ emotional reactions using the symptom grid, followed by introduction of techniques to prevent distress due to acute reactions to stress and to post-traumatic stress disorder. It was then explained that the EMDR support programme was intended to prevent and/or treat symptoms arising from participants’ experience of isolation and to allow them to maintain and develop their coping resources. The intervention was expected to be useful to cope not only with the quarantine period inside the nursing home, but also with the separation from their families, with a view to facilitating a smooth reunion and return to normality following the separation period.

Defusing, a standardized method used in rapid treatments and emergencies, was employed to give relevant information on post-traumatic stress reactions and to enhance individual coping resources. The butterfly hug was taught to develop participants’ awareness of their own emotional reactions. This technique (Artigas and Jarero, 2014; Jarero, 2020), generally used in the EMDR-IGTP protocol, involves patients using self-administering bilateral stimulation and it is used in stabilization, reinforcement of resources and safe place as well as for processing traumatic episodes.

One of the most recent publications on the Cost-effectiveness of psychological treatments for post-traumatic stress disorder in adults, Mavranezouli et al. (2020), report that EMDR is one of the most effective intervention for adults with PTSD and the most cost-effective, due to the brief duration. That is why EMDR was chosen and supported for this intervention with the healthcare workers in the middle of the COVID-19 Pandemic.

The three following meetings (meetings 2, 3, and 4) focused on the distress and the needs of the team and group EMDR (EMDR-IGTP) treatment protocol was carried out. Participants reported feelings of helplessness, being unable to see their loved ones and at the loss of their normal pre-COVID routines, feelings of absurdity but especially for the residents about missing Easter festivities and contact with their families due to isolation. They also reported a wide variety of traumatic memories. Some of the worst memories treated included seeing their elderly patients struggling to breathe, watching them die without being able to touch them, having to rush continually from room to room, the lack of space in the morgue for dead bodies and others that show the dramatic situation the healthcare workers experienced. In addition, somatic symptoms like difficulty sleeping, anxiety and worry and irritability and restlessness were also reported.

Sessions can range from 1 h to 1 h and 30 min. The participants were guided through a safe/secure place exercise or breathing exercises. The EMDR-IGTP leader asked them to think about the worst part of the event (the current crisis) and then to draw that image on the paper provided. They were then asked for the related Subjective Units of Disturbance (SUD) rating and told to write the corresponding number on their picture. After that they were asked to look at their picture and to provide their own alternating bilateral stimulation with the Butterfly Hug. The participants were then instructed to draw another picture of their own choice related to the event and rate it according to its level of distress. Processing continued with the adults looking at the second picture and using the Butterfly Hug. The process was repeated twice more so that each participant drew four pictures, and provided a SUD rating for each. The final level of distress associated with the current crisis was then assessed by asking to focus on the drawing that was most disturbing and to identify the current SUD level. This number was then written on the back of the paper and was the 5th SUD rating for the session. The participants then drew a final picture that represented their future vision of themselves, along with a word or a phrase that described that picture. No SUD rating was provided for this picture. The drawing and the phrase were then paired with the Butterfly Hug. The clients were instructed to close their eyes, scan their body, and do the Butterfly Hug or grounding techniques for the stabilization.

The last meeting (meeting 5) was conducted using the same procedure as the previous three, but some time was dedicated to post-test administration of the IES-R scale and the emotion thermometer and to allowing participants with EMDR to share their impressions of the experience. The intervention ended with concluding comments and a wrap up.

### Measurements

Pre- and post-intervention measurements were carried out in meetings 1 and 5, respectively, to verify results. A follow up measurement was carried out 9 months after the intervention.

The following questionnaires were administered to participants.

1. Impact of Event Scale-Revised (IES-R) in accordance with the criteria of the DSM IV-TR (Weiss and Marmar, 1997) validated and translated into Italian (Pietrantonio et al., 2003). This psychometric test consists of 22 items. It includes three subscales measuring the following dimensions: intrusion, avoidance, and hyperactivation. Participants were asked to rate their level of post-traumatic symptoms using a 5-point Likert scale ranging from 0 (= “not at all”) to 4 (= “a lot”) referring to the previous 7 days. The total score between 0 and 88. The cut-off of 33 highlights a high risk of PTSD; in line with the literature, there are no specific cut-offs for scale interpretations.
2. The emotion thermometer, a non-standardized instrument, used to measure on a scale of 1–10 participants’ subjective experience of six emotions and their consequences: stress, anxiety, depressed mood, anger, difficulty sleeping, and need for help.

## Results

### Qualitative results

The following is the coordinator’s summary of feedbacks given by the healthcare team.

The first topic is the most difficult moment the team had to face, i.e., “*We had to close the gates to visitors. It was the toughest moment for two reasons: the first was because we were gradually but surely realizing how serious the situation was; the second was the feeling that one of the cornerstones of our service, a “safe haven” was being removed: the constant presence of our residents’ family members. In our value, family members are an essential element: there have never been limits to visiting times or the duration of visits, and their presence is an important resource, that we are loosing with the Pandemic and that we have to cope without.”*

Regarding the EMDR intervention, the coordinator describes its efficacy as follows: “*EMDR interventions helped immensely to process the critical moments and to connect with each other. It enabled all of us to acknowledge our tiredness, our longing for our families, and the desire to get out of the nursing home*…*without making us feel less important, less efficient or less worthy!! It allowed us all to express our fragilities and transform them into resources! One of our colleagues compared the EMDR meetings to weaving silk: any of the single threads would break under the pressure of the loom and personal dynamics. EMDR brought all the threads together to make one strong, tenacious, resistant one.”*

### Descriptive statistics

**FIGURE 1:**
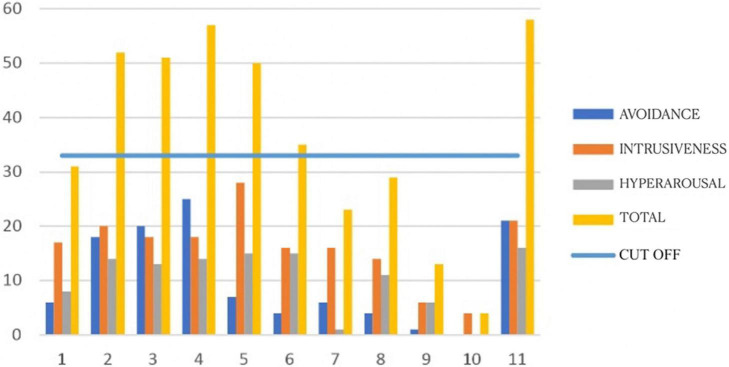
Impact of Event Scale-Revised (IES-R) pre-intervention scores for each participant.

**FIGURE 2:**
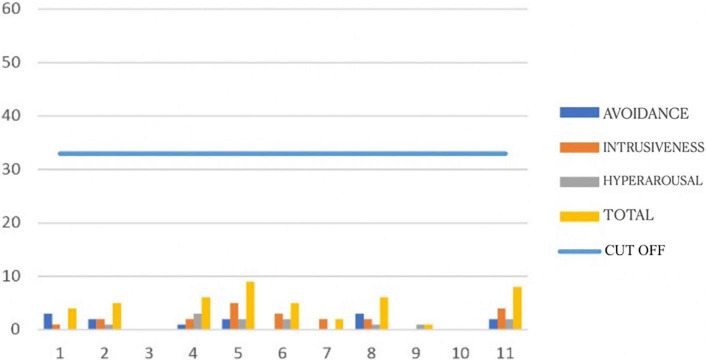
Post-intervention Impact of Event Scale-Revised (IES-R) scores for each participant.

The graphs in Figures 1, 2 show the symptoms scores obtained with the IES-R for each participant at meeting 1 and 5, before and after the EMDR programme. They show that all participants’ scores improved after the programme.

Table 1 shows average scores for the IES-R. It can be seen that pre-test scores were high for intrusiveness *M* = 16,18 (*SD* = 6,65), avoidance *M* = 10,18 (*SD* = 8,96), and hyperarousal *M* = 11,18 (*SD* = 6,11). All the average post-test scores are lower: avoidance *M* = 1,18 (*SD* = 1,25); intrusiveness *M* = 1,91 (*SD* = 1,64); hyperarousal *M* = 1,09 (*SD* = 1,04).

**TABLE 1:** Average scores, standard deviations, and significance level Impact of Event Scale-Revised (IES-R) (Weiss and Marmar, 1997).

	Avoidance	Intrusiveness	Hyperarousal	Total
**PRE**				
Mean	10.18	16.18	11.18	37.55
SD	1.18	1.91	1.09	4.18
**POST**				
Mean	8.96	6.65	6.11	18.36
SD	1.25	1.64	1.04	3.09
*p*<	0.005*	0.0000*	0.0001*	0.0000*

*Significance *p* < 0.05.

The difference between pre-and post-treatment average scores is statistically significant.

With regards to the results obtained with the emotion thermometer, shows that all participants reported a substantial improvement in all the dimensions following the EMDR treatment.

Overall, as Table 2 shows, participants scored high on the emotion thermometer subscales measuring psychosomatic disturbances, like stress (*M* = 7,36; *SD* = 2,73), anxiety (*M* = 6,73; *SD* = 3,52), sleeping problems (*M* = 7,91; *SD* = 2,51). The high average need for help score shows that the EMDR intervention would indeed potentially be very helpful.

**TABLE 2:** Emotion thermometer: Average scores, standard deviations, and significance level.

	Stress	Anxiety	Mood	Anger	Sleep	Help
**PRE**						
Mean	7.36	6.73	4.09	5.36	7.91	7.09
SD	2.73	3.52	3.62	4.30	2.43	2.51
**POST**						
Mean	3.64	3.64	2.27	2.09	3.91	3.36
SD	3.29	3.61	3.29	2.39	3.48	3.64
*p*<	0.0000*	0.0003*	0.005*	0.001*	0.0000*	0.001*

*Significance *p* < 0.05.

Average scores for the same dimensions were lower following the intervention. The difference between pre- and post-intervention scores is statistically significant.

**FIGURE 3:**
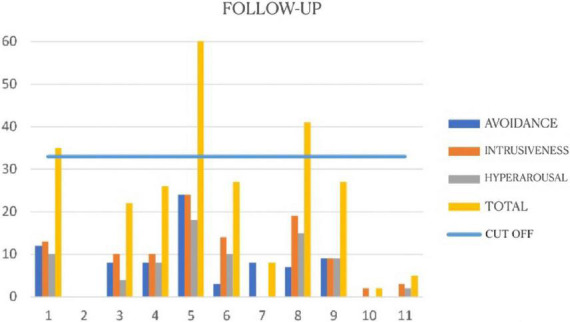
Follow-up Impact of Event Scale-Revised (IES-R) (Weiss and Marmar, 1997).

The IES-R questionnaire was administered to participants again at follow up, 9 months after the end of the intervention. The numbers obtained show a slight increase on all subscales: intrusiveness (*M* = 7,5; *SD* = 6,48), avoidance (*M* = 7,2; *SD* = 7,33), and hyperarousal (*M* = 7,1; *SD* = 7,01). This total increase (*M* = 21,8; *SD* = 19,83) may be attributable to the prolonged psychophysical stress caused by the continuing COVID emergency situation, and by the effect of the second wave of COVID-19 arrived in Italy in that time, as it reported in Figure 3.

## Discussion

Our results confirm that the isolation, that was a fundamental strategy during the COVID-19 pandemic was a disturbing event that had a strong emotional impact on the nursing home healthcare workers; and that EMDR was effective in contrasting this impact. However, due to the lack of a control group, it is not possible to exclude that any other type of intervention, interrupting the social isolation, would have been effective in reducing the effects of stress-induced PTSD.

Participants’ post-intervention IES-R scores were significantly lower on the intrusiveness, avoidance, and arousal subscales. Post-intervention scores on the emotion subscales also showed significant decreases in levels of stress, anxiety, depressed mood, anger, sleep problems, and perceived need for help. Although preliminary, these results are statistically significant and show that the EMDR programme we conducted was effective in reducing the effects of stress-induced PTSD as a result of isolation during the COVID-19 pandemic. The outcome was an improvement in the psychological wellbeing of participants, also confirmed in participant feedback. In addition to reducing the negative emotional charge of the event and the PTSD symptoms, it also improved the general emotional climate within our group.

Our results thus confirm that EMDR treatment can be applied in the short term and/or in emergency situations. The protocols and techniques can be used on their own or integrated into broader programmes with a wide variety of applications in individual or group settings.

Group EMDR seems to be a useful instrument for both prevention and treatment. It is of economic value because it lowers the cost of downstream management of mental health problems (de Bont et al., 2019). Our results are in line with those of other studies in showing that the standard EMDR protocol can be successfully adapted for use online to conduct EMDR therapy online (e.g., Fisher, 2021), including on workers involved in healthcare during the COVID-19 emergency (Pérez et al., 2020; Tarquinio et al., 2020). Still, they cannot be considered conclusive due to the small size of our treatment groups and the lack of a control group. Nevertheless, our aim was to report the outcome of a programme intended to prevent medium- and long-term disturbances, and to provide treatment for acute and chronic symptoms caused by the stress associated with the COVID-19 emergency situation in which the participants were currently immersed. In conclusion, future research involving larger samples and control groups is necessary to properly assess the effectiveness of group psychotherapy conducted online. Additionally, we recorded an increase in PTSD symptoms at the 9 month follow up, considering the different waves and the long duration of the Pandemic, even if not significant, suggesting that support spread over a wider period of time might be more effective in these circumstances.

Nevertheless, online administration of the EMDR protocol opens new possibilities in terms of timely intervention and prevention of long-term psychological disturbances as a result of acute stress.

## Data availability statement

The raw data supporting the conclusions of this article will be made available by the authors, without undue reservation.

## Ethics statement

Ethical review and approval was not required for the study on human participants in accordance with the local legislation and institutional requirements. The patients/participants provided their written informed consent to participate in this study.

## Author contributions

EF, MG, EG, IF, and MP contributed to conception and design of the study and wrote first draft of the manuscript. EF and MG organized the database. MP performed the statistical analysis. All authors wrote sections of the manuscript and contributed to manuscript revision, read, and approved the submitted version.
